# Development and validation of a diagnostic prediction model for pancreatic ductal adenocarcinoma: VAPOR 1, protocol for a prospective multicentre case–control study

**DOI:** 10.1136/bmjopen-2024-094505

**Published:** 2025-08-27

**Authors:** Caoimhe M Walsh, James Murray, Ivan Laponogov, Aaron Parker, James K Ellis, Valerio Converso, Emma Austin, Piers R Boshier, Charles Czajkowski, Duncan Spalding, Ahmed Al-Mukhtar, Adam E Frampton, Keith J Roberts, Sanjay Pandanaboyana, Chris Halloran, Eithne Costello, Hemant Kocher, Vikramjit Mitra, Zaed Hamady, Bilal Al-Sarireh, Samir Pathak, William Kyle Mitchell, Ajith Kumar Siriwardena, Claire Westlake, Stephen P Pereira, Ioannis Spiliotis, Sujata Biswas, VAPOR Collaborators, Patrik Španěl, Kirill Veselkov, Linda D Sharples, George B Hanna, VAPOR Collaborators, Pallavi Arya

**Affiliations:** 1 Department of Surgery and Cancer, Faculty of Medicine, Imperial College London, London, UK; 2 Department of Medical Statistics, Faculty of Epidemiology and Population Health, London School of Hygiene & Tropical Medicine, London, UK; 3 Patient and Public Involvement Representative, Pancreatic Cancer UK, London, UK; 4 Imperial College Healthcare NHS Trust, London, UK; 5 Sheffield Teaching Hospitals NHS Foundation Trust, Sheffield, UK; 6 Royal Surrey NHS Foundation Trust, Guildford, UK; 7 Section of Oncology, University of Surrey, Guildford, UK; 8 University Hospitals Birmingham NHS Foundation Trust, Birmingham, UK; 9 Newcastle Upon Tyne Hospitals NHS Foundation Trust, Newcastle Upon Tyne, UK; 10 Liverpool University Hospitals NHS Foundation Trust, Liverpool, UK; 11 Department of Molecular & Clinical Cancer Medicine, University of Liverpool, Liverpool, UK; 12 Barts Health NHS Trust, London, UK; 13 North Tees and Hartlepool NHS Foundation Trust, Hartlepool, UK; 14 University Hospital Southampton NHS Foundation Trust, Southampton, UK; 15 Swansea Bay University Health Board, Port Talbot, UK; 16 Leeds Teaching Hospitals NHS Trust, Leeds, UK; 17 Dorset County Hospital NHS Foundation Trust, Dorchester, UK; 18 Manchester University NHS Foundation Trust, Manchester, UK; 19 University Hospitals Plymouth NHS Trust, Plymouth, UK; 20 Royal Free Hospital Campus, University College London, London, UK; 21 Milton Keynes University Hospital NHS Foundation Trust, Milton Keynes, UK; 22 Buckinghamshire Healthcare NHS Trust, Amersham, UK; 23 Imperial College London, London, UK; 24 Department of Chemistry of Ions in Gaseous Phase, J Heyrovsky Institute of Physical Chemistry Czech Academy of Sciences, Praha, Czech Republic

**Keywords:** Case-Control Studies, Clinical Decision-Making, Pancreatic disease, Gastrointestinal tumours, Pancreatic surgery, Triage

## Abstract

**ABSTRACT:**

**Introduction:**

Pancreatic ductal adenocarcinoma (PDAC) continues to have extremely poor patient outcomes, unlike other cancer types which have seen significant improvements in their treatments and survival. A major contributing factor is that PDAC is often detected at an advanced, incurable stage. In the UK, nearly half of patients have stage 4 disease at the time of diagnosis, which has a profound effect on treatment options and, ultimately, survival. To address the challenge of early detection of PDAC, this study aims to develop and validate a clinical prediction model based on a non-invasive breath test. The proposed breath test aims to assist general practitioners in the triaging of patients who present with symptoms that do not meet current criteria for urgent suspected PDAC pathway referral.

**Methods and analysis:**

The Volatile organic compound Assessment in Pancreatic ductal adenOcaRcinoma (VAPOR 1) study is a prospective, multicentre, case–control study that aims to recruit 771 participants from England, Wales and Scotland. These include adult participants, aged ≥18 years, in three cohorts: pancreatic ductal adenocarcinoma; benign pancreatic controls (chronic pancreatitis or new-onset diabetes); healthy controls with a normal pancreas on imaging. A one-off breath sample will be obtained from participants who have fasted for at least 6 hours, and participant demographics and clinical data will be recorded. Breath samples will be analysed using gas chromatography–mass spectrometry to identify the volatile organic compounds (VOCs) present. Relationships between VOCs of interest and the presence of PDAC will be explored, and a clinical prediction model will be developed using statistical and machine learning methods and internally validated.

**Ethics and dissemination:**

The VAPOR 1 study has received approval from the South East Scotland Research Ethics Committee 02, and from the Health Research Authority and Health and Care Research Wales (REC 22/SS/0061). Results of this study will be published in open-access peer-reviewed journals, and disseminated through pancreatic cancer conference presentations. In addition, lay summaries shared on our website, social media platforms and through our charitable funder, Pancreatic Cancer UK, will enable engagement with patients and the wider public.

**Trial registration number:**

NCT05727020.

**STRENGTHS AND LIMITATIONS OF THIS STUDY**

- The study includes a benign cohort with new-onset diabetes to minimise potential confounding caused by the presence of diabetes in some patients diagnosed with pancreatic cancer.
- Volatile organic compound (VOC) analysis from breath samples follows a standardised process using validated gas chromatography–mass spectrometry methods.
- The study design is aligned with similar clinical studies investigating different gastrointestinal cancer types to facilitate the development of an integrated prediction model for early detection of multiple gastrointestinal cancers.
- Participant recruitment occurs in secondary care environments, while the ultimate test is intended for use in primary care.
- Participants receiving antibiotics within 8 weeks are excluded from the study, which could impact the application of the diagnostic prediction model in the clinical setting.

## Introduction

### Diagnostic challenges of pancreatic ductal adenocarcinoma

 Pancreatic ductal adenocarcinoma (PDAC) is the fifth most common cause of cancer-related deaths worldwide,^1^ and the incidence is expected to rise. Between 2006 and 2016, the number of deaths due to PDAC increased by 30%.^2^ In the UK, PDAC is the 10th most common type of cancer, but due to the challenges faced with early diagnosis and a lack of effective treatments for advanced disease, survival remains extremely poor. This makes it the fifth most common cause of cancer-related mortality in the UK (based on 2017–2019 data).^3^ PDAC is expected to become the second most common cause of cancer-related mortality in the USA by 2030.^4^

Pancreatic cancer does not usually cause symptoms in the early stages. The most common initial symptoms of PDAC are indigestion, loss of appetite, fatigue and ‘feeling different’, all of which are also common in patients without cancer.^5^ This makes early diagnosis of pancreatic cancer a significant challenge, as demonstrated by the longer time interval between first presentation in primary care and diagnosis, compared with other cancers.^6^ As a result, PDAC is frequently diagnosed at an advanced stage, when curative treatment options are limited.

The outlook for PDAC patients has improved only minimally over the last 50 years, with currently only 5% of those diagnosed surviving beyond 10 years, compared with 1% in the 1970s.^3^ The failure to improve survival of PDAC is largely a consequence of failure in the diagnostic pathway; therefore, early detection of PDAC remains an unmet clinical need.^7^

There is currently no screening programme available for pancreatic cancer in the UK general population, although the European Registry of Familial Pancreatic Cancer and Hereditary Pancreatitis trial screens high-risk genetically susceptible individuals.^8^ Thus, existing pathways for PDAC detection are based on referrals for patients either with symptoms or incidental findings of PDAC during investigations being undertaken for other reasons. These pathways currently use cross-sectional imaging (eg, CT, MRI) or endoscopic ultrasound to diagnose suspected PDAC, with pancreatic biopsy and histological confirmation for definitive diagnosis. The invasive nature of the gold standard diagnostic investigations, in addition to the non-specific symptoms associated with PDAC, underlies the challenges in diagnosis. For patients with non-specific symptoms, these invasive procedures and exposure to ionising radiation will frequently lead to over-investigation, as most patients will have benign pathology. In addition, patients are subject to unnecessary anxiety, and there are cost consequences to healthcare systems. In contrast, an overly cautious approach to referring patients with non-specific symptoms may result in missed or delayed cancer diagnoses. Therefore, alternative, more acceptable methods are required to help improve early detection of PDAC and reduce unnecessary testing.

### Proposed solution

A triage test will overcome the challenge of earlier detection of PDAC, by better targeting patients for referral to the appropriate specialised tests. Breath testing has ideal characteristics for a triage test. It is non-invasive, simple to undertake, relatively cheap, safe and universally acceptable to patients of all ethnicities.^9^ Such a test would directly benefit patients presenting with non-specific symptoms who do not meet existing criteria for referral. The proposed test is based on the detection of volatile organic compounds (VOCs), which are carbon-containing compounds that are sufficiently volatile to be detectable in the gas phase at room temperature and can be found in exhaled breath. Pilot studies in a range of cancers of the digestive system, including PDAC, have suggested that a VOC-based exhaled breath test may be sensitive and specific enough to identify patients at risk of PDAC, when used as a triage test.^10^

### Objectives

The objective of the VAPOR 1 (Volatile organic compound Assessment in Pancreatic ductal adenOcaRcinoma) study is to develop and internally validate a clinical prediction model (CPM) to triage the risk of PDAC based on profiles of VOCs obtained from the non-invasive breath test. The clinical utility of the model to enable referral for the appropriate reference test will be investigated, and the additional value of patient and clinical characteristics will be assessed. This will then be compared against the current standard practice for all patients presenting with symptoms indicative of pancreatic cancer.

## Methods and analyses

### Study design

VAPOR 1 is a UK prospective case–control study to construct a PDAC detection model based on VOCs in exhaled breath. The study involves the collection of breath at a single time point from eligible participants and is open to all National Health Service (NHS) trusts and diabetes education centres within the UK. The VAPOR 1 study is sponsored by Imperial College London and has obtained NIHR Clinical Research Network portfolio adoption to support study recruitment.

### Study duration

The VAPOR 1 study commenced in December 2022 and is due to be completed by August 2025.

### Definitions of study groups

VAPOR 1 has a case–control study design, with three groups of participants: PDAC, benign conditions and healthy controls.

PDAC: participants who either have a confirmed diagnosis of PDAC according to a biopsy or tissue sample taken from the pancreas, or who are due to undergo surgical resection for suspected PDAC (with histological confirmation to follow within 3 months).

Benign pancreatic conditions: participants with either new-onset diabetes mellitus (diagnosed within the previous 6 months) or chronic pancreatitis diagnosed clinically or radiologically at any time. Diabetes mellitus is confirmed if the participant’s average circulating blood sugar level, determined by glycated haemoglobin, is greater than 48 mmol/mol at the time of diagnosis.

Negative control group: participants with a radiologically normal pancreas confirmed by abdominal imaging, ideally within the previous 12 months.

### Eligibility

Patients aged ≥18 years will be eligible for VAPOR 1 if they satisfy one of the three group definitions earlier.

Exclusion criteria are: previous treatment (surgery, chemotherapy or radiotherapy) for confirmed PDAC; active infection, antibiotics or immunosuppressive medication within the preceding 8 weeks; any cancer in the previous 5 years; surgery altering the anatomy of the upper gastrointestinal tract (eg, Roux-en-Y gastric bypass, Whipple’s procedure); pregnancy; unable to provide a breath sample; unable to provide written informed consent.

### Sample size

The sample size of 205 PDAC cases was based on a target sensitivity of 85%, with 7% precision and 80% power, informed by pilot data on 132 patients.^10^ We aim to recruit the same number of each type of control, totalling 615 participants with complete data. Allowing for 20% attrition due to loss of samples, we will recruit 771 participants, which includes at least 256 with cancer.

Riley *et al*^11^ also recommend ensuring that the sample size achieves small mean absolute prediction error (MAPE), defined as the mean absolute difference between predicted and observed outcomes (cancer=1, no cancer=0), and large variation explained by the CPM, measured by Cox-Snell R^2^, which ranges from 0 for no predictive value to 1 for perfect prediction. Additionally, the area under the receiver-operating curve (AUC) provides an estimate of the ability of the CPM to discriminate between cancer and non-cancer cases. The resulting CPM based on 615 participants will have an MAPE of 0.048, estimate cancer incidence with precision 0.088, and achieve a minimum Cox-Snell R^2^ of 0.431 based on forty predictors. The SE in the AUC achieved by the CPM will be 0.016, based on an AUC of 0.86.

### Participant recruitment

Participants will be identified as potentially eligible for inclusion in VAPOR 1 by their direct care teams at participating NHS hospital sites and diabetes education centres across England, Scotland and Wales. PDAC participants will be identified through HepatoPancreaticoBiliary multidisciplinary team meetings in addition to operating theatre, endoscopy, imaging or outpatient clinic lists, or emergency presentations to the hospital. Patients with new-onset diabetes mellitus will be identified by direct care teams, including diabetes specialist nurses and referrals from general practice. Those with chronic pancreatitis will be identified from hospital outpatient clinic, imaging or endoscopy lists and will be confirmed by imaging within no strict timeframe. Healthy controls will be identified through review of operating theatre, endoscopy, imaging and outpatient clinic lists, ensuring potential participants have undergone imaging confirming a normal pancreas within the last year. Patients in other research studies are eligible if requisite consent is given and the study has ethical approval. Potential participants will be approached about study participation by a member of their local VAPOR 1 research team. If they are interested in participating, they will be provided with the participant information sheet for the study. Where possible, participants will be given at least 24 hours to decide whether or not to take part in this study. In all instances, participants will be given the maximum time available to decide whether to take part in this study. Participant recruitment visits will be aligned with planned hospital appointments, investigations or procedures wherever possible. Fully informed written consent will be obtained prior to obtaining their breath sample, which is collected as two breath samples given at one time point.

### Breath collection

**Figure 1:**
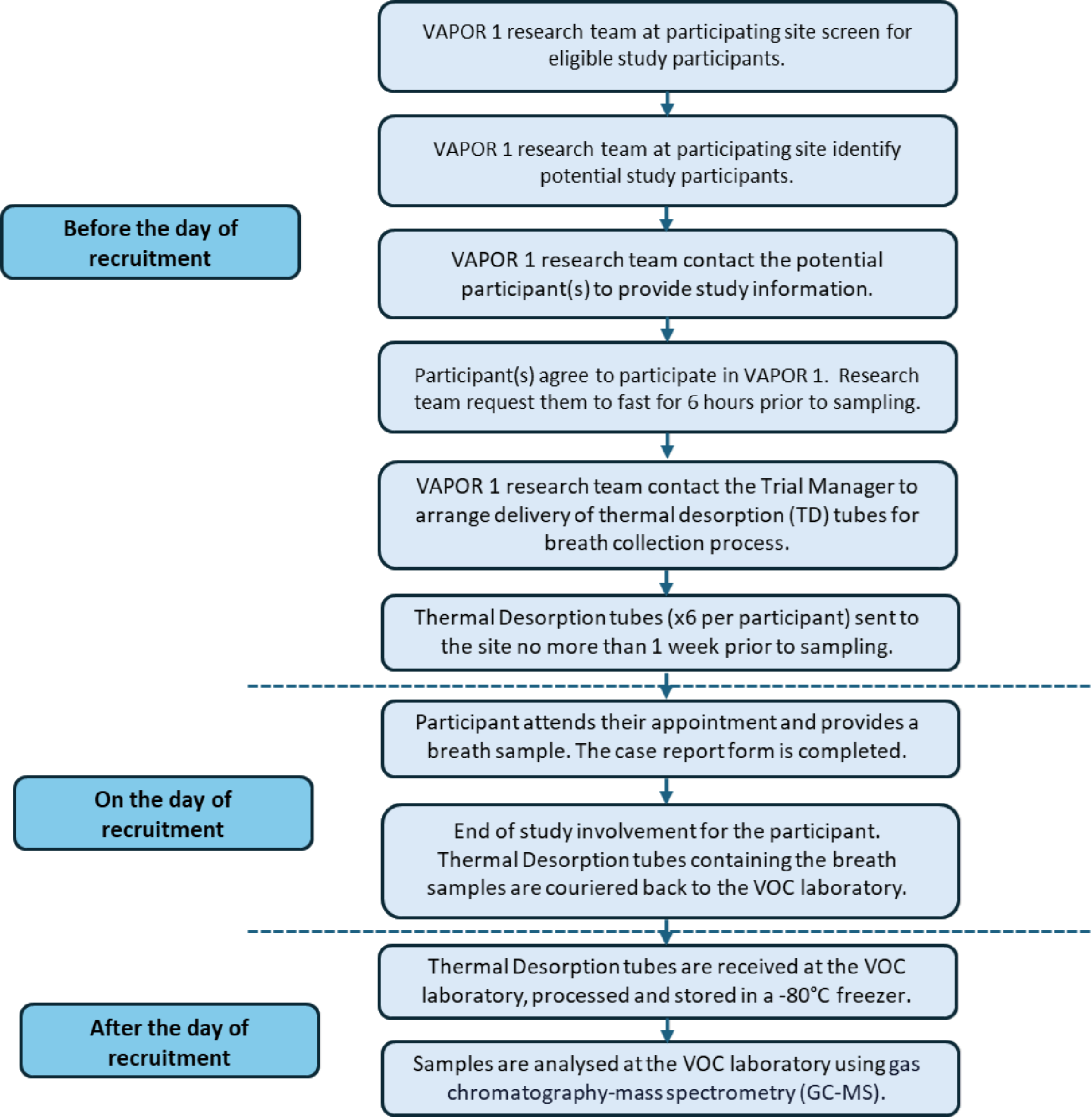
A flowchart demonstrating VAPOR 1 study procedures before, during and after recruitment. VAPOR 1, Volatile organic compound Assessment in Pancreatic ductal adenOcaRcinoma; VOC, volatile organic compound.

All research team members who will be recruiting participants are trained in breath collection by an approved member of the Imperial College London VOC Laboratory. Participants are asked to fast, apart from water, for a minimum of 6 hours prior to breath collection. For participants undergoing surgery or a procedure, breath samples must be obtained preoperatively, prior to administration of sedation, anaesthetic or other pharmacological agents. After providing written informed consent, the research team will collect demographics, tumour characteristics and medical history from the participant (see online supplemental table 1). They will then be asked to rinse their mouth with water and provide breath samples by exhaling into two single-use breath collection bags via a mouthpiece that is subsequently sealed. A bespoke pump developed in-house will collect breath from each bag onto two thermal desorption (TD) tubes for 4 min, at a rate of 200 mL/min,^12^ resulting in four TD tubes per patient, each containing 800 mL of breath. Room air samples will also be collected onto two additional TD tubes to control for potential background environmental contamination. All TD tubes are transported at room temperature to the Imperial College London VOC Laboratory. Other factors that could affect VOC profiles, such as dietary intake and environmental exposures, including smoking, have been considered in the study design. As a result, participants are requested to fast for 6 hours prior to breath collection, with the time the participant last ate and drank recorded as well as the last cigarette and alcohol intake, if any. Figure 1 shows the study procedures before, during and after recruitment.

### VOC analysis

Breath and room air samples will be analysed at the Imperial College London VOC Laboratory in accordance with established standard operating procedures (SOPs). On receipt of the samples, the TD tubes are purged with dry, inert gas to remove moisture and stored at −80°C until analysed. Volatolomic profiling is undertaken using thermal desorption–gas chromatography–time of flight–mass spectrometry (TD-GC-ToF-MS) instruments (Markes TD100-XR, Agilent 6890, BenchTOF2, SepSolve, UK) equipped with either a Rxi-624SilMS column (RESTEK, UK) for the ‘mid-polar’ method or Stabilwax-DA column (RESTEK) for the ‘polar’ method. Two breath samples and a room air sample will be analysed using the ‘mid-polar’ method and the remaining breath and room air samples will be analysed using the ‘polar’ method. For further identification of VOCs, samples can be recollected and analysed by two-dimensional TD-GCxGC-ToF-MS (Markes TD100-XR, Agilent 7890, Sepsolve modulator, BenchTOF2, SepSolve).

Deuterated internal and surrogate standards are added to each TD tube to account for variation in sampling and analysis. Regular system suitability checks and quality control samples are run on each gas chromatography–mass spectrometry (GC-MS) instrument to ensure the validity of results. The acquired spectra will be compared against the National Institute of Standards and Technology mass spectral library by matching mass spectra-retention indices and authentic standards for chemical verification of compounds. Data will be extracted, pre-processed and quality controlled using ChromSpace (Markes) and in-house scripts.

Scientists analysing the GC-MS data will have access to pseudonymised data. All study data will be collected according to the Data Protection Act 2018 and in line with the General Data Protection Regulation (GDPR). To ensure data minimisation, the data that will be collected has been carefully decided by the researchers and statisticians for the study to ensure only the relevant and suitable data is collected for the intended purposes. Study data that leave the local clinical/research teams will be converted to a linked pseudonymised format and will be stored electronically within an online password-protected database. Access to the data will be restricted to authorised users and will be controlled and stored in accordance with the Data Protection Act 2018 and GDPR. No identifiable patient data will leave the local clinical/research team. Study data transferred to external organisations will be sent in a pseudonymised format and will be transferred via Imperial College’s approved secure data transfer system. Data Sharing Agreements will be in place prior to any data sharing.

### Statistical analysis

The number of patients recruited into the VAPOR 1 study, breath samples provided, and samples successfully processed and analysed will be reported in a flow diagram. The number (%) of cases where there was failure to provide breath samples, quality control failures, exclusion from the study following recruitment or withdrawals of informed consent will be reported, along with summaries of baseline characteristics by study group. Missing data will be reported for each variable. We will report all findings from the study according to the Transparent Reporting of a multivariable prediction model for Individual Prognosis or Diagnosis Statement.^13^

#### Developing and reporting the CPM

Initially, relationships between candidate VOCs and the presence of PDAC will be explored using univariate logistic regression models, including linear, polynomial and cubic splines.

Given the large number of initial variables, a screening step will be undertaken to identify the most promising candidate VOCs for inclusion in statistical models, with approximately the top 50 being retained. To develop the CPM, a logistic regression model with variable selection and importance assessment techniques will be fitted. Robust variable selection methods will be used, specifically the least absolute shrinkage and selection operator (LASSO) and elastic net. LASSO aids in selecting a more parsimonious model by shrinking less important predictor coefficients to zero, while elastic net combines the penalties of LASSO and Ridge regression, effectively managing correlated predictors.^14^

Elastic net is particularly beneficial when dealing with highly correlated variables, as it can select groups of related predictors, making it well-suited for multivariate datasets like VOC profiles. The best-performing model, determined through nested cross-validation (CV), will be advanced for further analysis.

In addition to the statistical methods, random forest and deep neural networks (DNNs) will be used to enhance model predictions. Random forest constructs multiple decision trees from different data subsets and combines their predictions, handling complex, non-linear relationships in the VOC data and identifying key VOCs that predict PDAC. DNNs, capable of capturing patterns in high-dimensional data, will be employed to model non-linear relationships in the VOC data. The DNN architecture will include a designed number of hidden layers and neurons, with regularisation techniques like dropout applied to prevent overfitting.

The model will be enhanced by assessing the additional diagnostic value of patient and clinical features (eg, family history of cancer, presenting symptoms), potentially improving its predictive power and clinical applicability.

A 10-fold CV will be used for internal validation of statistical and machine learning models. Nested CV will be applied to best identify the penalty terms in LASSO and elastic net models, as well as to fine-tune the hyperparameters in random forest and DNNs, reducing the risk of overfitting across all models. For each fold in the internal CV, the sensitivity, AUC, precision-recall AUC and the MAPE will be calculated and summarised by median (IQR) values across folds. Apparent performance with 95% CIs and optimism will be calculated by 100 bootstrap resamples. Predictors chosen in ≥50% bootstrapped selection routines will be taken forward.

For the final model, bias-corrected ROC (receiver operating characteristic) curves and calibration plots will be provided. All model coefficients with SEs, and ORs with 95% CIs will be reported.

#### Outlier and missing data considerations

Since very high values of some VOCs may be indicative of PDAC, measurements considered extreme will be assessed by study clinicians/scientists without knowledge of the study group. Any excluded values will be documented and reported.

The primary analysis will use complete data. If individual VOCs are missing, we will assume ‘missing at random’ conditional on cancer status and all other predictors.^15^ If indicated by the missing data patterns, a sensitivity analysis will be conducted, imputing missing VOCs using chained equations to produce 20 datasets. Imputation models will be developed for cases and controls separately and will be consistent with the substantive logistic model. CPM development will be repeated for each imputed dataset and a final set of predictors will be identified. Using this final set, CPMs will be fitted for each imputed dataset and the results will be combined using the Rubin rules.^16^

#### Derivation of diagnostic rules

A clinical decision rule will be derived from probabilities of PDAC generated by the final CPM, with thresholds for referral for definitive testing, review within 6 months and discharge.

Development of decision rules will consider the cost of missing a PDAC case and of incorrectly declaring PDAC in the control group. The net benefit of the CPM will be calculated across a range of candidate thresholds.^17^ We will use decision curve analysis^18^ to compare the net benefit of the proposed CPM against the current standard of care of referring all symptomatic patients. We will also consider the threshold which maximises the expected number of patients taking the test before misdiagnosis occurs, weighted by the ‘clinical cost’ of a false negative relative to a false positive.^19^ This relative ‘clinical cost’ will be elicited from expert clinicians and members of the study steering group, which includes patient representatives.

#### Software for statistical analysis

Auto-deconvolution of GC-MS data will be performed using MSHub solution (V.0.1 or higher).^20^ All data cleaning and analyses will be conducted using R (V.4.3.3 or higher) or Python (V.3.12.9 or higher) as required.

### Ethics and dissemination

#### Ethical considerations

The VAPOR 1 study has received approval from South East Scotland Research Ethics Committee (REC) 02 on 12 October 2022, and from Health Research Authority and Health and Care Research Wales on 17 October 2022.

#### Safety considerations

The study has been assessed by the clinical trial manager to be low risk to participants, similar to their usual standard of care. This assessment was undertaken using risk assessment tool templates, in accordance with Imperial College London Research Governance and Integrity Team SOPs. The non-invasive breath test exposes participants to minimal risk. All adverse events considered to be related to the collection of the breath samples will be reported to the VAPOR study team via the adverse event reporting form (as a paper copy or on the REDCap study database). Serious adverse events will be reported to the VAPOR study team within 24 hours of the site becoming aware of the event, and will be reported to the Sponsor and REC, as required by the Imperial College London Research Governance and Integrity Team’s SOPs.

#### Data collection and management considerations

Prior to providing a breath sample, eligibility criteria will be assessed and recorded by the research team member, including dates and results of eligibility tests. Participant demographics and baseline lifestyle factors, medications, medical history, family history of cancer and symptoms will be recorded in the VAPOR REDCap database. This is a secure online database with access limited to the research staff trained on study procedures and authorised on the study delegation log. Data monitoring will be conducted through both central monitoring of the REDCap database and on-site monitoring visits to conduct source data verification. Breath samples will be processed according to our standardised laboratory workflow which ensures full traceability of participants’ breath samples by logging recorded data on our Laboratory Information Management System. Participants’ VOC profiles will be obtained via mass spectrometry during breath sample analysis, and this will be recorded alongside their clinical data (see online supplemental table 1 which lists the data collected). Where a participant withdraws consent, this will be recorded on the REDCap database. All data and samples collected up to the point consent was withdrawn will be kept for analysis. No further breath samples will be obtained; however, data related to existing samples may still be collected.

### Dissemination plan

The results of the VAPOR 1 study will be presented at national and international conferences. Authorship for any publications of this research will follow the International Committee of Medical Journal Editors guidance. Links to the publication will be shared in the relevant registries. A lay summary of the results will be developed with patient and public involvement (PPI) representatives, and this will be shared on the study website and social media accounts. The lay summary will also be made available to participants who request it via their recruiting centre and will be disseminated by relevant charities.

### Patient and public involvement

Patient representatives from Pancreatic Cancer UK were involved in developing the study, including creating a plan to ensure patient and public access to the research and contributing to the content of the lay summary. They strongly supported the development of a non-invasive breath test for pancreatic cancer and stated it would be preferable to other interventional tests. Feedback from a PPI panel from the NIHR London In Vitro Diagnostic Co-operative was used to create the patient-facing documents for the study. A patient representative continues to advise on public engagement throughout the study, and also engages with the research teams at participating VAPOR 1 sites, through events such as the VAPOR study collaborators’ meeting. The Trial Steering Committee includes an independent lay member with lived experience of pancreatic cancer to ensure decisions in all aspects of the study consider the viewpoint of patients and the public.

## Supplementary material

10.1136/bmjopen-2024-094505 online supplemental table 1
