# Supplementary material for: Development and validation of a diagnostic prediction model for pancreatic ductal adenocarcinoma: VAPOR 1, protocol for a prospective multicentre case–control study
Source: BMJ Open. 2025 Aug 27;15(8):e094505. doi: 10.1136/bmjopen-2024-094505 (PMC12406849; doi:10.1136/bmjopen-2024-094505)
Supplement: online supplemental table 1 [file bmjopen-15-8-s001.pdf]

Supplementary Table 1. Data collected from participants recruited to the VAPOR 1 study

Variable Category	Predictors (type)
**Demography and lifestyle**	
	Gender at birth (binary)<br>If female, any current pregnancy<br>Age at enrolment (continuous)<br>Height in metres (continuous)<br>Weight in kilograms (continuous)<br>Body Mass Index (calculated from height and weight)<br>Ethnicity (categorical: Arab, Asian or Asian British – Bangladeshi, Asian or Asian British – Indian, Asian or Asian British – Pakistani, Chinese, Other Asian background, Mixed – Asian and White, Mixed – Black African and White, Mixed – Black Caribbean and White, Black or Black British – African, Black or Black British – Caribbean, Other Black background, Other Mixed background, Gypsy or traveller, White, Other White background, Other Ethnic background, Prefer not to say)<br>Smoking status (categorical: Never/Ex-/Smoker or Vaper) and if available the number of cigarettes per day (continuous), hours since last smoke/ vape (continuous), and if ex-smoker number of pack years (continuous)<br>Alcohol consumption (binary) and if available the number of units per week (continuous) and time since alcohol consumed (continuous)
**Study group**	
	Which group the patient was recruited to (categorical: PDAC, control with benign conditions, control with normal pancreas)<br>Additional information for PDAC participants if available:<br>- Date of diagnosis<br>- Method of diagnosis (categorical: endoscopic biopsy (EUS) or pancreatic resection specimen).<br>- Site of the tumour within pancreas (categorical: head, neck, body, tail, uncinate process)<br>- Tumour differentiation (categorical: well-, moderately-, poorly-differentiated)<br>- Summary / Conclusion from histology report<br>- TNM Stage (ordinal: Stage I-IV)<br>- Modality used for TNM staging (categorical: pathological or radiological)<br>- Site of any metastatic disease<br>- Surgery performed to remove the tumour<br><br>Additional information for chronic pancreatitis participants if available:<br>- Year of diagnosis<br>- Aetiology (categorical: alcohol, recurrent/ severe acute pancreatitis, idiopathic, hereditary, medication, other)<br>- Date of most recent abdominal imaging and type of imaging (categorical: CT, MRI, Ultrasound, other)<br>- Pancreatic enzyme replacement therapy use (binary)<br><br>Additional information for new-onset diabetes participants, if available:<br>- Year of diagnosis<br>- Method of diagnosis (categorical: symptomatic, routine diabetes screening test, incidental finding, other)<br>- Most recent HbA1c result and date of test<br>- Diabetes control method (categorical: diet, medication, other)<br><br>Additional information for healthy control participants, if available:<br>- Date of most recent abdominal imaging

	- Type of scan (categorical: CT, MRI, Ultrasound, Other)
**Patient symptoms**	
	Reason for initial referral from GP (categorical: Jaundice, weight loss, abdominal pain, back pain, nausea/vomiting, diarrhoea, constipation, new-onset diabetes, other, or no symptoms).<br>Additional information if available:<br>- Duration of symptoms (continuous)
**Medical history**	
	History of cancer(s) > 5 years ago (binary)<br>Additional information if available:<br>- Site(s) (categorical)<br>- Year(s) of diagnosis (continuous)<br>Previous treatment for cancer (chemotherapy, radiotherapy or surgery)<br>Systemic infection within the last 8 weeks<br>Previous pancreatic surgery, or surgery altering the anatomy of the upper gastrointestinal tract<br>Other medical conditions (categorical: Hypertension, type I/II diabetes, liver disease, gallstones, asthma, COPD, heart disease, kidney disease, coeliac disease, inflammatory bowel disease, or other)
**Blood results**	
	Result available (binary), if so, date of test and test results for:<br>- Bilirubin<br>- CA19-9 tumour marker<br>- CEA tumour marker
**Concomitant medications**	
	Currently taken medication (all binary, one patient can have many):<br>- Proton pump inhibitor,<br>- NSAIDs,<br>- Anticoagulant/antiplatelet,<br>- Diabetic medication,<br>- Antibiotics,<br>- Laxatives,<br>- Steroids,<br>- Immunosuppressants,<br>- Other.
**Family history of cancer**	
	At least one relative with history of pancreatic cancer (binary)<br>Additional information if available:<br>- Relation to patient (categorical: parent, grandparent, etc.)
**Breath sampling details**	
	Date and time of breath sampling<br>Location of breath sampling (categorical: outpatient department, endoscopy ward, ward, theatres, anaesthetic room, etc.)<br>Time of last food, fluid and alcohol (if applicable)<br>Fasting status of the participant (binary)<br>Breath collection device number<br>File number<br>Thermal desorption tube number
**Laboratory results**	
	Profiles of VOCs (all continuous)
