# Supplementary material for: Development and validation of a diagnostic prediction model for pancreatic ductal adenocarcinoma: VAPOR 1, protocol for a prospective multicentre case–control study
Source: BMJ Open. 2025 Aug 27;15(8):e094505. doi: 10.1136/bmjopen-2024-094505 (PMC12406849; doi:10.1136/bmjopen-2024-094505)
Supplement: Supplementary file 2 [file bmjopen-2024-094505.reviewer_comments.pdf]

## PEER REVIEW HISTORY

BMJ Open publishes all reviews undertaken for accepted manuscripts. Reviewers are asked to complete a checklist review form and are provided with free text boxes to elaborate on their assessment. These free text comments are reproduced below.

### ARTICLE DETAILS

**Title (Provisional)**

Development and validation of a diagnostic prediction model for pancreatic ductal adenocarcinoma: VAPOR 1, protocol for a prospective multicentre case-control study.

**Authors**

Walsh, Caoimhe M; Murray, James; Laponogov, Ivan; Parker, Aaron; Ellis, James K; Converso, Valerio; Austin, Emma; Boshier, Piers R; Czajkowski, Charles; Spalding, Duncan; Al-Mukhtar, Ahmed; Frampton, Adam E; Roberts, Keith J; Pandanaboyana, Sanjay; Halloran, Chris; Costello, Eithne; Kocher, Hemant; Mitra, Vikramjit; Hamady, Zaed; Al-Sarireh, Bilal; Pathak, Samir; Mitchell, William Kyle; Siriwardena, Ajith Kumar; Westlake, Claire; Pereira, Stephen P; Spiliotis, Ioannis; Biswas, Sujata; VAPOR Collaborators, .; Španěl, Patrik; Veselkov, Kirill; Sharples, Linda; Hanna, George B

---

### VERSION 1 - REVIEW

---

| | |
|---|---|
| **Reviewer** | **1** |
| **Name** | **Liao, Zhuan** |
| **Affiliation** | **Second Military Medical University, Department of Gastroenterology, Changhai Hospital** |
| **Date** | **24-Jul-2025** |
| **COI** | **None** |

---

This study aims to develop an early diagnostic model for pancreatic cancer based on exhaled breath volatile organic compound (VOC) profiling, representing a clinically significant research direction. The manuscript presents substantial content with coherent structure and logical flow. Below are recommendations for enhancement:

1.Page 8, line 46: The mention of external validation lacks corresponding cohort description elsewhere in the manuscript. It is recommended to provide details of this external validation cohort.

2.Page 10, lines 36-37: The exclusion criterion "active infection" requires more precise definition. Given established literature documenting that oral infectious conditions (e.g., periodontal diseases) produce confounding VOCs (e.g., $H_2S$), consideration should be given to excluding volunteers with active oral infections or history of such conditions—even in remission. At minimum, this information should be incorporated into Supplemental Table 1.

3.Chronic pancreatitis diagnostic criteria should be explicitly stated.

4.Page 12, lines 46-47: Subjects were only instructed to fast (water permitted) for >6 hours before breath collection. Additional pre-sampling restrictions should be implemented regarding smoking, alcohol consumption, and oral hygiene procedures.

5.Page 18, lines 45-46: The software version number must be explicitly stated.

---

## VERSION 1 - AUTHOR RESPONSE

**Reviewer 1: Dr. Zhuan Liao, Second Military Medical University**

Comments to the Author:
This study aims to develop an early diagnostic model for pancreatic cancer based on exhaled breath volatile organic compound (VOC) profiling, representing a clinically significant research direction. The manuscript presents substantial content with coherent structure and logical flow. Below are recommendations for enhancement:

**1. Page 8, line 46: The mention of external validation lacks corresponding cohort description elsewhere in the manuscript. It is recommended to provide details of this external validation cohort.**

Response:

Thank you for raising this. This sentence was referring to a separate, future study (VAPOR2) intended to occur following the completion of the VAPOR1 study (to validate its findings). Therefore to avoid confusion we have removed this sentence, since it does not relate to a cohort that is part of the VAPOR1 study [page 8 of marked copy document, INTRODUCTION - Objectives].

**2. Page 10, lines 36-37: The exclusion criterion "active infection" requires more precise definition. Given established literature documenting that oral infectious conditions (e.g., periodontal diseases) produce confounding VOCs (e.g., $H_2S$), consideration should be given to excluding volunteers with active oral infections or history of such conditions—even in remission. At minimum, this information should be incorporated into Supplemental Table 1.**

Response:

Thank you for your comments which we agree with. The exclusion criterion "active infection" refers to all types of infection, including oral infectious conditions. As mentioned in the 'Eligibility' section of the protocol, this refers to any such infection occurring within the preceding eight weeks (even if the participant is no longer infectious / in remission at the time of breath sampling). [page 10 of marked copy document, METHODS AND ANALYSES – Eligibility].

**3. Chronic pancreatitis diagnostic criteria should be explicitly stated.**

Response:

Thank you for highlighting this. This is a multi-centre study and based on our feasibility assessments, it was apparent that chronic pancreatitis is managed differently across the various hospital sites (in some centres it is managed by the surgical team, in others by the medical gastroenterology team). In some instances patients are given a radiological diagnosis based on CT, MRI or EUS features, and in other instances patients are given a clinical diagnosis. We therefore accept either a clinical or

radiological diagnosis, and this is reported for each participant on the VAPOR REDCap database (see Supplementary Table 1). We have now also specified this in the manuscript for clarification. [page 10 of marked copy document, METHODS AND ANALYSES – Definitions of study groups].

**4. Page 12, lines 46-47: Subjects were only instructed to fast (water permitted) for >6 hours before breath collection. Additional pre-sampling restrictions should be implemented regarding smoking, alcohol consumption, and oral hygiene procedures.**

Response:

Thank you for highlighting this important point. Regarding pre-sampling oral hygiene procedures, our group has previously undertaken a study (Vadhwana B, Belluomo I, Boshier PR, Pavlou C, Španěl P, Hanna GB. Impact of oral cleansing strategies on exhaled volatile organic compound levels. Rapid Commun Mass Spectrom. 2020 May 15;34(9):e8706. doi: 10.1002/rcm.8706) which demonstrated that an oral water rinse was the optimum oral cleansing strategy prior to breath sampling, hence we include an oral water rinse as part of the breath collection procedure, immediately before the participant provides a breath sample. As a result, we chose not to impose any more oral hygiene restrictions on participants pre-sampling. [page 12 of marked copy document, METHODS AND ANALYSES – Breath collection].

Regarding smoking and alcohol, participants are not permitted to consume alcohol during the 6 hour fast prior to breath sampling. We chose not to impose any further restrictions beyond this time period in an effort to maintain conditions as close to real life as possible, however as mentioned in the protocol we document all participants' smoking and alcohol history (where relevant), including time of last cigarette and time of last alcohol consumption prior to breath sampling. This allows us to take these factors into account during our data analysis. [page 13 of marked copy document, METHODS AND ANALYSES – Breath collection].

**5. Page 18, lines 45-46: The software version number must be explicitly stated.**

Response:

Thank you for this. Given that this is a study protocol detailing planned future data analysis, rather than publication of results/data analysis already undertaken, we have now included the current version numbers our team use and stated that we will use this version or higher, to allow for any potential significant software updates between now and when the analysis is undertaken that our team wishes to avail of. For the final publication of our results, we will of course specify explicitly which software (including version numbers) we have used. [page 18 of marked copy document, METHODS AND ANALYSES – Statistical analysis].
